# A model bridging waterlogging, stomatal behavior and water use in trees in drained peatland

**DOI:** 10.1093/treephys/tpac037

**Published:** 2022-04-06

**Authors:** Che Liu, Qian Wang, Annikki Mäkelä, Hannu Hökkä, Mikko Peltoniemi, Teemu Hölttä

**Affiliations:** Department of Forest Sciences, Faculty of Agriculture and Forestry, University of Helsinki, Latokartanonkaari 7, P.O. Box 27, Helsinki 00014, Finland; Institute for Atmospheric and Earth System Research (INAR), University of Helsinki, Latokartanonkaari 7, P.O. Box 27, Helsinki 00014, Finland; Department of Landscape Architecture, College of Architecture, Changsha University of Science and Technology, Chi-Ling Street 45, 410076 Changsha, Hunan, China; Department of Forest Sciences, Faculty of Agriculture and Forestry, University of Helsinki, Latokartanonkaari 7, P.O. Box 27, Helsinki 00014, Finland; Institute for Atmospheric and Earth System Research (INAR), University of Helsinki, Latokartanonkaari 7, P.O. Box 27, Helsinki 00014, Finland; Natural Resources Institute Finland (Luke), Chi-Ling Street 45, 410076 Changsha, Hunan, Finland; Natural Resources Institute Finland (Luke), Chi-Ling Street 45, 410076 Changsha, Hunan, Finland; Department of Forest Sciences, Faculty of Agriculture and Forestry, University of Helsinki, Latokartanonkaari 7, P.O. Box 27, Helsinki 00014, Finland; Institute for Atmospheric and Earth System Research (INAR), University of Helsinki, Latokartanonkaari 7, P.O. Box 27, Helsinki 00014, Finland

**Keywords:** hydraulic conductance, Scots pine, semi-process-based modeling, soil flooding, stomata

## Abstract

Waterlogging causes hypoxic or anoxic conditions in soils, which lead to decreases in root and stomatal hydraulic conductance. Although these effects have been observed in a variety of plant species, they have not been quantified continuously over a range of water table depths (WTD) or soil water contents (SWC). To provide a quantitative theoretical framework for tackling this issue, we hypothesized similar mathematical descriptions of waterlogging and drought effects on whole-tree hydraulics and constructed a hierarchical model by connecting optimal stomata and soil-to-leaf hydraulic conductance models. In the model, the soil-to-root conductance is non-monotonic with WTD to reflect both the limitations by water under low SWC and by hypoxic effects associated with inhibited oxygen diffusion under high SWC. The model was parameterized using priors from literature and data collected over four growing seasons from Scots pine (*Pinus sylvestris* L.) trees grown in a drained peatland in Finland. Two reference models (RMs) were compared with the new model, RM1 with no belowground hydraulics and RM2 with no waterlogging effects. The new model was more accurate than the RMs in predicting transpiration rate (fitted slope of measured against modeled transpiration rate = 0.991 vs 0.979 (RM1) and 0.984 (RM2), *R*^2^ = 0.801 vs 0.665 (RM1) and 0.776 (RM2)). Particularly, RM2’s overestimation of transpiration rate under shallow water table conditions (fitted slope = 0.908, *R*^2^ = 0.697) was considerably reduced by the new model (fitted slope = 0.956, *R*^2^ = 0.711). The limits and potential improvements of the model are discussed.

## Introduction

Waterlogging, or flooding in soil characterized by high soil water content (SWC) and shallow water table depth (WTD), drives oxygen from soil pores and leads to hypoxia or anoxia (i.e. insufficient or absent oxygen) in the belowground parts of plants. This phenomenon inhibits aquaporin activity and mitochondrial respiration and causes cytosol acidosis in the roots of nearly all trees, thereby significantly decreasing root hydraulic conductance (Lambers et al. 2008, Parent et al. 2008, Kreuzwieser and Rennenberg 2014). Following the inhibited water uptake in roots, a variety of tree species showed stomatal closure in waterlogged conditions (Pezeshki and Chambers 1986, Pezeshki et al. 1996, Ferner et al. 2012). This consequent decrease of stomatal conductance occurs likely because of whole-plant hydraulic signaling for maintaining water homoeostasis (Jackson 2002, Christmann et al. 2013) and is more severe in flood-sensitive individuals or species (Pezeshki et al. 1996, Pearson et al. 2013, Kreuzwieser and Rennenberg 2014). Ultimately, severe symptoms may occur, including leaf necrosis, bark shedding and whole-tree dieback, and the tree’s production and growth (above- and belowground) may be strongly impaired (Parolin 2001, Alaoui-Sossé et al. 2005, Ferry et al. 2010, Kreuzwieser and Rennenberg 2014, Repo et al. 2020).

Despite their wide occurrence and drastic consequences, the waterlogging effects on whole-tree hydraulics have not been clearly quantified due to difficulties in conducting continuous observations of their whole hydraulic system. Nevertheless, the similar responses of stomatal behavior to excess soil water and to air/soil dryness suggest that the existing models of root and stomatal conductance may be modified to represent waterlogging effects. More specifically, the recent observation of the similar impacts of drought and flooding on root conductance has suggested the possibility of simulating the effects of both disturbances in similar mathematical structures (Domec et al. 2021). Such a model, once verified by data, should describe the soil–tree–atmosphere continuum more accurately on a wider range of water conditions, and shed light upon whole-tree hydraulics and leaf gas exchange responding simultaneously to above- and belowground factors in a quantitative manner.

Among the models of stomatal behavior in relation to air dryness, irradiance and temperature, the Lagrangian optimization model is one of the most widely tested and successful (Cowan 1977, Cowan and Farquhar 1977, Hari et al. 1986, Mäkelä et al. 1996, Hari and Mäkelä 2003). The core of this type of model is that the steady state of stomatal behavior realizes the optimization (maximization) of the integrated difference between carbon gain and water cost over a given time, i.e. ${\rm{max}} \int \big(A(t)-\lambda E(t)\big)\mathrm{d}t$ (Hari et al. 1986), which yields the marginal water-use efficiency (MWUE, *λ*) at the steady state. In later studies, the model has been developed to cover more physiological processes and/or wider scales (Dewar 2002, Katul et al. 2010, Medlyn et al. 2011, Manzoni et al. 2013, Prentice et al. 2014, Bell et al. 2015) and used for quantifying specific ecophysiological issues, such as effects of severe drought, trees’ competition for water and global functional diversity of plants (Lin et al. 2015, Wolf et al. 2016, Lu et al. 2020). Nonetheless, the relationship between soil water and MWUE in the related variants of the model is always monotonic, and excess water (i.e. waterlogging or flooding) turning into a constraint to soil-to-root and stomatal conductance has not been incorporated. Not only is such an incorporation important for quantifying the ecophysiological processes, but it should also improve our ability to account for the effects of increasing extreme events of precipitation on stomatal behavior in large-scale predictions (Palmer and Räisänen 2002, Christensen and Christensen 2003, Frei et al. 2006, IPCC 2021).

Considering such potential benefits and previous studies, we hypothesized that the impact of waterlogging in decreasing trees’ water uptake rate is similar in magnitude to that of air/soil dryness. In other words, similar algebraic expressions should be employed to describe the effects of both waterlogging and air/soil dryness on soil-to-root conductance, water use and stomata behavior. To test the hypothesis, we linked the Lagrangian optimal stomata model with a soil-to-leaf conductance model (Duursma et al. 2008, Nikinmaa et al. 2013, Hölttä et al. 2017, Dewar et al. 2018), which was modified to describe the waterlogging effects on water use in trees. This bridging was constructed by writing MWUE, instead of a single parameter *λ*, as a segmented (and thus ‘non-monotonic’) function of SWC and soil-to-leaf conductance affected by WTD. The underlying assumption of the decreased conductance was that the inhibited oxygen diffusion under hypoxic conditions reduces soil-to-root conductance, which was implemented in an algebraically simplified manner. The model was tested using data of sap flow density in Scots pine (*Pinus sylvestris* L.) trees, WTD and other environmental factors collected from a subarctic drained peatland site in the Finnish Lapland over four growing seasons.

## Materials and methods

### Study site and data acquisition

The study site was in Sattasuo (66°30′ N, 26°42′ E, 165 m above sea level), a subarctic flat peatland drained by open ditch in the Finnish Lapland. The mean air temperature in July over 1981–2010 was 15.0 °C, and mean precipitation sum 505 mm per year (Stenberg et al. 2018). Typically, the ground was snow-covered from October through May. The first drainage was conducted in 1934, and the latest one before the studied period (2008–11) was in 2006. In 2006, the standing volume of the dominant Scots pine (*Pinus sylvestris* L.) was 91 m^3^ ha^−1^. The site was classified as medium in productivity (Vasander and Laine 2008, Stenberg et al. 2018).

Six naturally grown Scots pine trees (aged 66–87 years, counted at the breast height) were chosen as sample trees, of which two (Nos 2 and 3) were noticeably taller than the other four (Table 1). To determine their sapwood thickness without damaging them, another eight Scots pine trees of similar size at the site were bored, and sapwood thickness $\big({\vartheta}_{\mathrm{SW}},\mathrm{mm}\big)$ measured on the cores was correlated with diameter at breast height (DBH, *d*, mm) as

(1) \begin{equation*} {\vartheta}_{\mathrm{SW}}=0.0001\ {d}^2+0.1668\ d+9.755 \end{equation*}

${\vartheta}_{\mathrm{SW}}$ of the six sample trees was calculated by Eq. (1) (Table 1). Their sap flow was measured using thermal dissipation probes (TDPs; Granier 1987) through the unfrozen season (May to October) of 2008–11. A pair of TDP, comprising a heated (HP) and a reference (RP) probe, both *c.* 3.5 cm long, were installed on the north side of each sample tree’s stem at the height of *c*. 1.3 m. The HP was heated with a constant power of 0.2 W (Lu et al. 2004), and the RP was directly below HP at a distance of *c*. 10 cm. All the probes were covered by aluminium foils with ventilation holes. The voltage difference between HP and RP (Δ*U*) was measured every 30 s, recorded as 10-min average, and converted to sap flow density using Baseliner 4.0 (Oishi et al. 2016). The sap flow signals of trees nos 2 and 5 were extremely noisy since June 2011, and thus their data were discarded thereafter.

**Table 1:** Information of the scots pine sample trees. DBH, diameter at breast height; ${J}_{\mathrm{max}}^{(O)}$, maximum observed sap flow density over 2008–11; SW, sapwood. DBH and height were measured in 2006. SW thickness was estimated by Eq. (1).

Tree number	1	2	3	4	5	6
DBH (cm)	12.9	16.2	15.0	15.7	13.3	17.5
SW thickness (cm)	3.29	3.94	3.70	3.84	3.37	4.20
Height (m)	11.5	14.0	13.4	11.8	11.2	11.5
${J}_{\mathrm{max}}^{\big(\mathrm{O}\big)}$ (mol H_2_O m^−2^ SW s^−1^)	2.116	1.415	1.230	1.948	1.877	2.072

On a forest clearing 190 m north from the study site, air temperature, humidity (CS215 probe, Campbell, UT, USA) and photosynthetic photon flux density (PPFD; SP1110 pyranometer, Campbell) were recorded every 10 min. The vapor pressure deficit (VPD, in Pa) was calculated as

(2) \begin{equation*} \mathrm{VPD}=611\ {\rm{exp}} \left(\frac{17.502\ T}{T+240.97}\right)\left(1-{h}_{\mathrm{r}}\right) \end{equation*}

(Campbell and Norman 1998) where *T* is air temperature (°C), and *h*_r_ relative humidity. For the consistency with the process model (Eq. (4)), VPD was converted to the unit of mol H_2_O m^−3^ using the ideal gas law:

(3) \begin{equation*} D=\frac{\mathrm{VPD}}{8.3145\ \left(T+273.15\right)} \end{equation*}

To obtain the daily-level data for the subsequent analyses, the median of the largest 10% data by ranking of each day was gathered for each of the variables.

Evenly distributed in the site, 10 1-m-long tubes were mounted into the ground for manually measuring water table depth (WTD). The manual measurement was conducted at an interval of 1–2 weeks through the same period as of the other data. A water height meter (Intech Instruments ltd New Zealand) was installed at the approximate center of the site, recording WTD and water temperature (WTT) every hour. The meter’s temporal pattern of WTD was used for interpolating between the manual measurement data points to obtain the hourly WTD at each measurement tube. As for the other variables, the median of the largest 10% data by ranking of each day was taken as the daily WTD for the subsequent analyses.

### The hierarchical model

A hierarchical model was constructed, which comprises two levels, namely, process and data models. The process model is an optimal stomata model associated with a module on the dependence of soil-to-root conductance on WTD via SWC. The data model contains the probability density function (PDF) of the error between modeled (*E*^(M)^) and observed (*E*^(O)^) transpiration rate.

#### Optimal stomata model

In the optimal stomata model, the stomatal behavior maximizes the difference between the integrated carbon gain and water loss over a given time (Cowan and Farquhar 1977, Hari et al. 1986). At the steady state, transpiration rate (*E*^(M)^) is modeled proportional to the stomatal conductance for water (${g}_{\sigma}$, m s^−1^), which in turn is dependent on VPD (*D*), respiration rate (*R*, mol CO_2_ m^−2^ s^−1^) and PPFD (*I*, mol m^−2^ s^−1^)

(4) \begin{equation*} {E}^{\left(\mathrm{M}\right)}=1.6\ D\,{g}_{\sigma}=1.6\ D\left(\sqrt{\frac{C_{\mathrm{a}}-\frac{R}{f(I)}}{1.6\lambda D}}-1\right)f(I) \end{equation*}

where *λ* is MWUE (mol CO_2_ mol^−1^ H_2_O), *C*_a_ the atmospheric CO_2_ concentration (mol m^−3^), 1.6 the ratio of water vapor to CO_2_ diffusion rates, and *R* and *f*(*I*) (irradiance reaction curve), respectively, are modeled as follows (Hari and Mäkelä 2003, Mäkelä et al. 2004):

(5) \begin{equation*} R={\rm{max}} \left\{{R}_0{Q}_{10}^{T_{\mathrm{l}}/10},0\right\} \end{equation*}

where *T*_l_ is leaf temperature (Eq. (9)), *Q*_10_ relative increase of *R* per 10 °C, *R*_0_ the value of *R* at 0 °C; and

(6) \begin{equation*} f(I)=\frac{\iota \gamma I}{\iota I+\gamma } \end{equation*}

where *γ* is the saturation (asymptote) of the curve (m s^−1^), and *ι* (initial slope, m^3^ mol^−1^) is

(7) \begin{equation*} \iota ={\rm{max}} \left\{c\left(S-{S}_0\right),0\right\} \end{equation*}

where *c* is the slope coefficient (m^3^ (mol °C)^−1^), and *S* is the photosynthetic acclimation of foliage to temperature above the threshold (*S*_0_), of which the marginal change with respect to time (day) follows:

(8) \begin{equation*} \frac{\mathrm{d}S}{\mathrm{d}t}=\frac{T_{\mathrm{l}}-S}{\tau } \end{equation*}

where τ is the time constant (day) of the acclimation, and *T*_l_, leaf temperature, was calculated from air temperature (*T*, °C) and as

(9) \begin{equation*} {T}_{\mathrm{l}}=T+1.5\times{10}^3\ I \end{equation*}

(Kolari et al. 2007), where *I* is as in Eqs (4) and (6). The ‘S’-model (Eqs (6–8)) has been widely applied to physiological and growth acclimation to temperature and irradiance at a range of temporal and spatial scales (Suni et al. 2003, Mäkelä et al. 2004, Mäkelä et al. 2008, Schiestl-Aalto et al. 2015).

#### Water use and soil-to-leaf conductance

Different from evaluating MWUE as a parameter (*λ* in Eq. (4)) directly (Hari and Mäkelä 2003, Mäkelä et al. 2004, Liu et al. 2020), we modeled *λ* as a function of soil-to-leaf conductance (*k*_sl_, mol H_2_O m^−2^ leaf s^−1^ Pa^−1^), which has two components in series, i.e. soil-to-root (*k*_sr_) and root-to-leaf (*k*_rl_) conductance, following

(10) \begin{equation*} {k}_{\mathrm{sl}}^{-1}={k}_{\mathrm{sr}}^{-1}+{k}_{\mathrm{rl}}^{-1} \end{equation*}

When stomatal aperture is optimized and only constrained by nonstomatal factors (viz. carboxylation capacity and/or mesophyll conductance), *k*_sl_ and *λ* are inversely correlated (Dewar et al. 2018), which can be expressed empirically as

(11) \begin{equation*} {\rm{log}}_{10}\lambda ={z}_0+{z}_1{\rm{log}}_{10}\left(\frac{k_{\mathrm{sl}}}{k_0}\right) \end{equation*}

(${z}_1<0$; Hölttä et al. 2017) where *k*_0_ is the base-case xylem conductance (mol H_2_O m^−2^ leaf Pa^−1^ s^−1^), and *z*_0_ and *z*_1_ are parameters evaluated in this study.

The tree-specific root-to-leaf conductance (*k*_rl_ in Eq. (10)) was assumed constant through the study period and estimated as

(12) \begin{equation*} {k}_{\mathrm{rl},i}=\frac{{\rm{max}} \left\{{J}_{ij}\right\}}{\rho \left|{\Psi}_{\mathrm{lmin}}\right|} \end{equation*}

where ${\rm{max}} \big\{{J}_{ij}\big\}$ is the maximum sap flow density of tree *i* observed over the study period (i.e. data points *j*; Table 1), *ρ* leaf-to-sapwood area ratio, and $\big|{\Psi}_{\mathrm{lmin}}\big|$ the absolute value of the lowest leaf water potential (Duursma et al. 2008). The trees were assumed isohydric through the study period, and thus Ψ_lmin_ was assumed constant at −2 MPa, a typical value for Scots pine (Martínez-Vilalta et al. 2009). The other component of *k*_sl_, *k*_sr_ (Eq. (10)) was designed in a segmented manner to reflect its non-monotonic dependence on soil water content (SWC, *θ*, m^3^ m^−3^).

#### Soil-to-root conductance and water table depth

We segmented the soil-to-root conductance (*k*_sr_ in Eq. (10)) into increasing, stable and decreasing phases with respect to SWC (calculated from WTD; Eq. (17)) to reflect the switch from water (deep WTD) to oxygen (shallow WTD) limitation via a relatively stable stage. In the increasing phase of SWC, *k*_sr_ is a power function of SWC relative to its saturation (*θ*_sat_), i.e.

(13) \begin{equation*} {k}_{\mathrm{sr}}^{+}\left(\theta \right)={\xi}_m{\left(\frac{\theta }{\theta_{\mathrm{sat}}}\right)}^{\xi_p} \end{equation*}

where *ξ_m_* (mol m^−2^ s^−1^ Pa^−1^) is a parameter depending on root length index (m root m^−2^ ground surface), rhizosphere and fine root radii, and saturated soil conductivity, and *ξ_p_* is related to the soil water retention curve (Duursma et al. 2008, Nikinmaa et al. 2013). This form of correlation is also supported experimentally (Poyatos et al. 2018) at low to medium SWC.

We were not aware of any experimental studies on the responses of mature Scots pine to excess water featured with a gradient of SWC or WTD, of which the results would help hypothesize a continuous ${k}_{\mathrm{sr}}^{-}$ for the decreasing phase. However, the flooding-sensitive loblolly pine (*P. taeda* L.) has shown similar declines in root conductance under drought and flooding treatments compared with control (Domec et al. 2021). Thus, we assumed ${k}_{\mathrm{sr}}^{-}\big(\theta \big)$ to be also a power function with the base modified to represent the available soil porosity not occupied by water (i.e.$1-\theta /{\theta}_{\mathrm{sat}}$). Furthermore, to utilize prior knowledge on the optimal WTD (δ^*^; Hökkä et al. 2021), we introduced the optimal SWC (θ^*^) corresponding to δ^*^ (Eq. (17)) as a parameter. Hence,

(14) \begin{equation*} {k}_{\mathrm{sr}}^{-}\left(\theta \right)={\eta}_m{\left(\frac{2{\theta}^{\ast }-\theta }{\theta_{\mathrm{sat}}}\right)}^{\eta_p} \end{equation*}

where *η_m_* and *η_p_* are parameters. This formula resembles the soil-type-specific diffusivity coefficient of oxygen (Moldrup et al. 1996, 1997). The optimal conductance $\big({k}_{\mathrm{sr}}^{\ast}\big)$ was assumed to be maintained through a range of 20 cm of WTD between the increasing and decreasing phases, i.e.

(15) \begin{equation*} {k}_{\mathrm{sr}}^{\ast}\left(\theta \right)=\frac{k_{\mathrm{sr}}^{+}\left[\theta \left({\delta}^{\ast }+10\right)\right]+{k}_{\mathrm{sr}}^{-}\left[\theta \left({\delta}^{\ast }-10\right)\right]}{2} \end{equation*}

where $\theta \big(\bullet \big)$ is SWC as a function of WTD (Eq. (17)). Finally, over the range of the WTD data,

(16) \begin{equation*} {k}_{\mathrm{sr}}={\rm{min}} \left\{{k}_{\mathrm{sr}}^{+},{k}_{\mathrm{sr}}^{-},{k}_{\mathrm{sr}}^{\ast}\right\} \end{equation*}

Throughout this study, all values of *θ* were calculated from WTD (*δ*, cm) by

**Figure 1.**
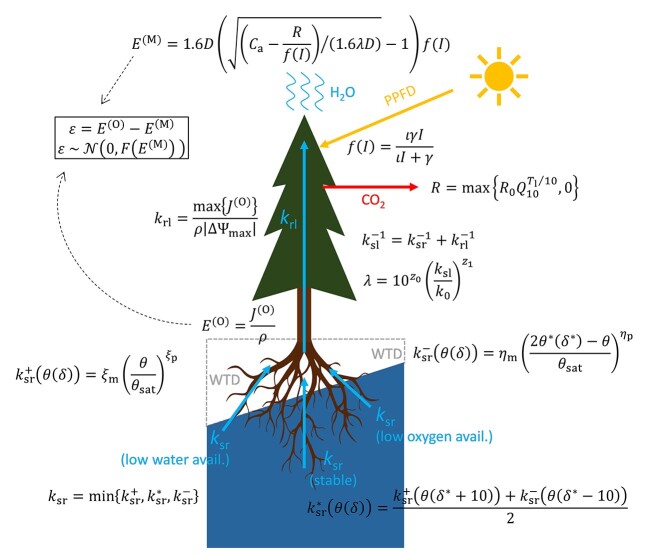
Model scheme. PPFD, photosynthetic photon flux density; WTD (*δ*), water table depth; avail., availability. See the main text for detailed explanation of the equations, Table 2 for meanings and prior ranges of the evaluated parameters, and Table S1 available as Supplementary data at *Tree Physiology* Online, for the full list of symbols.

(17) \begin{equation*} \theta ={\theta}_{\mathrm{res}}+\frac{\theta_{\mathrm{sat}}-{\theta}_{\mathrm{res}}}{{\left[1+{\left(0.072\ \delta \right)}^{1.371}\right]}^{1-{1.371}^{-1}}} \end{equation*}

where *θ*_res_ is the residual SWC (Leppä et al. 2020). From Eqs (4) through (17), transpiration rate and stomatal conductance are linked with WTD via MWUE in trees (Figure 1).

#### Data model and parameterization

The data model or the PDF of the error between tree-specific observed and modeled transpiration rate is

(18) \begin{equation*} {\varepsilon}_{ij}={E}_{ij}^{\left(\mathrm{O}\right)}-{E}_{ij}^{\left(\mathrm{M}\right)}={J}_{ij}/\rho -{E}_{ij}^{\left(\mathrm{M}\right)} \end{equation*}

where *i* is tree number, *j* the index of data points in the time series, *J_ij_* and *ρ* same as in Eq. (12), and ${E}_{ij}^{(\mathrm{M})}$ follows Eq. (4). Judged by the initial runs using the parameter priors, the error *ε_ij_* calculated by Eq. (18) was assumed to follow the normal distribution, of which the standard deviation (SD) parameter was correlated with *E*^(M)^ for the observed heteroscedasticity. This correlation was exponential (i.e. semi-log linear) in two trees (nos 4 and 6, noted collectively W_**1**_) and simple linear without significant intercept in the other four (no. 1–3 and 5, noted collectively W_**2**_). Thus, the likelihood function of ***ε*** is

(19) \begin{align*} & \mathrm{Prob}\left(\boldsymbol{\varepsilon} \right)=\prod_{i\in{\mathbf{W}}_{\mathbf{1}}}\prod_{j=\text{1}}^{N_i}\mathcal{N}\left(0,{\rm{exp}} \left(\alpha +{\beta}_{{\mathbf{W}}_{\mathbf{1}}}{E}_{{\mathbf{W}}_{\mathbf{1}},j}^{\left(\mathrm{M}\right)}\right)\right) \nonumber \\ & \times \prod_{i\in{\mathbf{W}}_{\mathbf{2}}}\prod_{j=1}^{N_i}\mathcal{N}\left(0,{\beta}_{{\mathbf{W}}_{\mathbf{2}}}{E}_{{\mathbf{W}}_{\mathbf{2}},j}^{\left(\mathrm{M}\right)}\right) \end{align*}

where ***ε*** is the error matrix $\big(\boldsymbol{\varepsilon} \stackrel{\scriptscriptstyle\mathrm{def}}{=}\big\{{\varepsilon}_{ij}\big\}\big)$, *α* and *β*, respectively, are the intercept (if used) and slope coefficients in the semi-log or simple linear regression for the SD parameters, *i* and *j* as in (Eq. (18)), *N_i_* the total number of data points of tree *i*, and the subscripts denote the ordinal numbers of the trees.

As ${k}_{\mathrm{sr}}^{+}$ has been found correlated with tree height (Martínez-Vilalta et al. 2007*a*), its multiplier coefficient (*ξ_m_* in Eq. (13)) was tree specific, each tree still sharing the same prior range (Table 2). Considering the possible difference among trees in the acclimation to temperature regarding reaction to irradiance (Eq. (7)), *c* was also tree specific with the same prior range. All the other parameters were shared by all the trees, including *η_m_* and *η_p_* (Eq. (14)) due to limited observed evidence. The model had 22 estimated parameters (including 19 for the process model; Table 2) and four direct input variables *D*, *I*, *T*_l_ and *δ*. All the parameters were assumed independently and uniformly distributed. The full list of symbols is attached in Supplementary data (Table S1 available as Supplementary data at *Tree Physiology* Online).

**Table 2:** Meanings, prior ranges and uses of the evaluated parameters of the process model.

	Meaning (unit, if applicable)	Prior range (minimum, maximum)	Equation	Ref.^1^
*ξ_mi_* ^2^	Multiplier (*ξ_m_*, mol m^−2^ s^−1^ Pa^−1^) and power (*ξ_p_*) coefficients of the correlation between soil water content and soil-to-root conductance during its increase	0.07, 0.15	13	1–3^3^
*ξ_p_*		7, 10	13	1–3^3^
*η_m_*	Similar to *ξ_m_ ξ_p_* but during the decrease of soil-to-root conductance due to excess water	0.05, 0.17	14	1–3^3^
*η_p_*		1.5, 10	14	4
*δ* ^*^	Optimal water table depth (cm)	20, 50	14, 15	5
*z*_0_	Intercept (*z*_0_) and slope (*z*_1_) of the log–log linear regression correlating marginal water-use efficiency and soil-to-leaf conductance	−5.2, −3.8	11	6–8
*z*_1_		−1.0, −0.5	11	6–8
*c_i_* ^2^	Slope of acclimation of leaves to temperature (m^3^ (mol C°)^−1^)	0.01, 0.07	7	9^4^
*γ*	Saturation of irradiance reaction in stomatal conductance (m s^−1^)	1.6 × 10^−3^, 3.0 × 10^−3^	6	8, 9

^1^References (Ref.): 1. Päivänen (1973); 2. Duursma et al. (2008); 3. Nikinmaa et al. (2013); 4. Moldrup et al. (1997); 5. Hökkä et al. (2021); 6. Hölttä et al. (2017); 7. Hari and Mäkelä (2003); 8. Liu et al. (2020); 9. Mäkelä et al. (2004).

^2^ *ξ_mi_* and *c_i_* are the tree-specific parameters in the process model, where *i* denotes the ordinal number of tree (1–6, Table 1).

^3^ *ξ_mi_* and *ξ_p_* are products of multiple parameters in Refs 2 and 3, and the reference range of each component parameter was used for calculating the priors of *ξ_mi_* and *ξ_p_* with adjustment based on Ref. 1 specifically on peatland. The prior ranges of *η_m_* and *η_p_* were similar to those of *ξ_mi_* and *ξ_p_*, respectively, but slightly broadened due to lack of direct observation in literature.

^4^ *c* is denoted *c*_1_ in Ref. 9, which estimated its values using shoot-level data. Considering the possible heterogeneity at whole-crown level, we broadened its prior range for the current study.

The maxima a posteriori (MAP) estimates of the parameters were sought by adaptive Markov chain Monte Carlo (MCMC) algorithm DREAM_(ZS)_ (Vrugt et al. 2009) in R 4.0.3 (R Development Core Team 2020) with package ‘BayesianTools’ (Hartig et al. 2019). Convergence was defined at $\hat{R}<1.1$, and only the second halves of the chains (i.e. without ‘burn-in’) were used for the convergence diagnostic and the subsequent analyses (Gelman et al. 2013). The 95% credible interval of predictive uncertainty was generated by Eq. (19), defined as the interval between the 2.5 and 97.5% quantiles of *ε* calculated with 6000 parameter vectors randomly sampled from the posterior distribution.

### Model performance assessment

To assess the improvement brought about by the new module linking MWUE to WTD with waterlogging effects (Eqs (10–17)); hereafter termed full model, FM), two reference models were calibrated for comparison: (i) the base optimal stomata model (Eqs (4–9)) with *λ* estimated directly as a temporally constant tree-specific parameter (RM1), and (ii) a bridged model including the soil-to-leaf conductance but with a monotonic correlation between *k*_sr_ and SWC (i.e. without considering waterlogging effects; RM2). The prior range of *λ* in RM1 was according to previous studies by similar models on Scots pine (Hari and Mäkelä 2003, Mäkelä et al. 2004, Liu et al. 2020). The heteroscedasticity descriptions in the data models (Eq. (19)) of RMs were adjusted accordingly. Otherwise, FM and RMs shared the same structure and prior ranges of parameters, MCMC method and input data.

The models’ overall performances were judged by linear regression with zero intercept of *E*^(O)^ to *E*^(M)^ computed with the parameters’ MAP estimates (hereafter simply *E*^(M)^). To compare the performances of FM and RM2 particularly on shallow WTD, this linear regression was performed with *E*^(M)^ using the full data as well as those in the range of ${k}_{\mathrm{sr}}^{-}$ (corresponding to $\mathrm{WTD}<{\delta}^{\ast }-10\ \mathrm{cm}$; Eq. (15)).

The tree-specific performance of FM was assessed by the normalized root-mean-square error (NRMSE) of the corresponding tree, defined as

(20a) \begin{equation*} {\mathrm{RMSE}}_i=\sqrt{\frac{\sum_{{j}=\mathbf{1}}^{{{N}}_{{i}}}{{\varepsilon}}_{{i}{j}}^{{2}}}{{{N}}_{{i}}}} \end{equation*}

(20b) \begin{equation*} {\mathrm{NRMSE}}_i=\frac{{\mathrm{RMSE}}_i}{\overline{E_i^{\left(\mathrm{O}\right)}}}\times 100 \end{equation*}

where $\overline{E_i^{(\mathrm{O})}}$ denotes the mean of *E*^(O)^ of tree *i* over the study period, and the rest of the notation is the same as in Eqs (18) and (19). To clarify the possible causes of the residual, correlation coefficient (Pearson’s *r*) was calculated between FM’s residual ($\varepsilon ={E}^{(\mathrm{O})}-{E}^{(\mathrm{M})}$, mol H_2_O m^−2^ leaf s^−1^) and WTT. The WTT was measured within the same tubes for the manual measurement of WTD. All the statistical analyses were performed in R 4.0.3.

## Results

**Figure 2.**
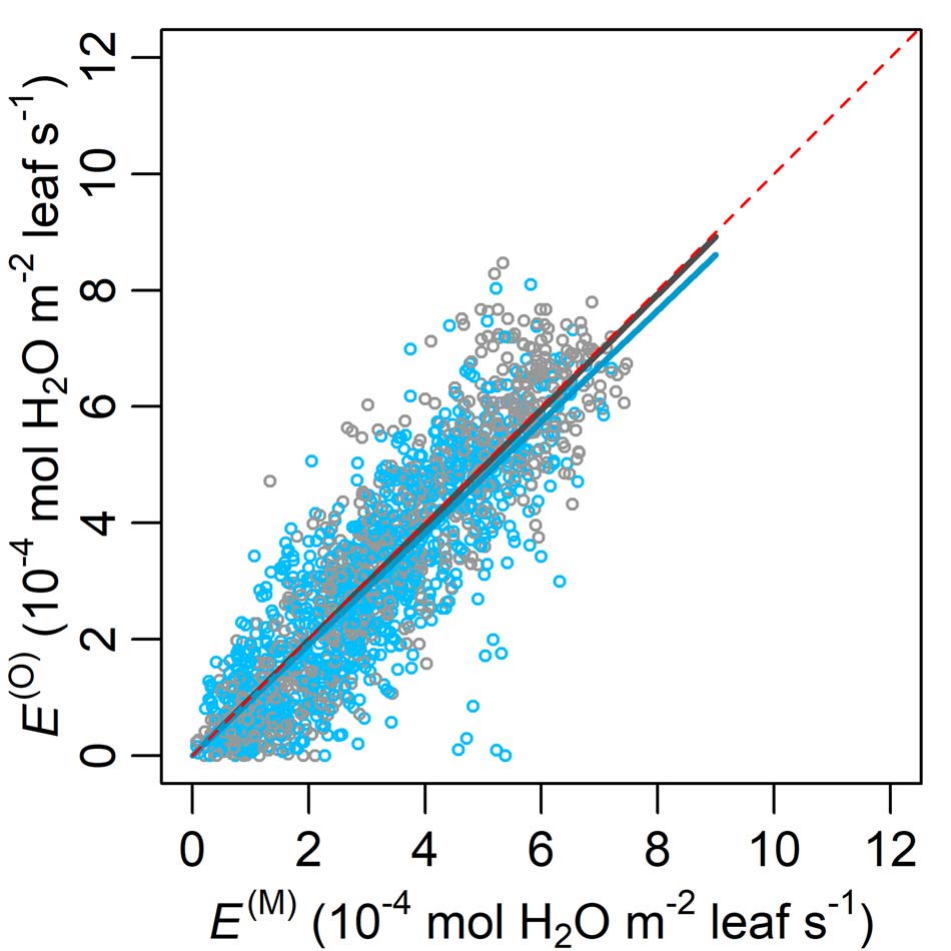
Overall performance of the full bridged model with waterlogging effects (FM). Lines without intercept were fitted to the data of observed transpiration rate (*E*^(O)^, converted from measured sap flow density per sapwood area; *y*) and simulated results (*E*^(M)^; *x*). Dark gray and dark blue lines, respectively, denote the fittings using the full data (light gray circles) and those with shallow WTD ($<{\delta}^{\ast }-10\ \mathrm{cm}=37.98\ \mathrm{cm}$; Table 3; blue circles). Their respective equations are *y* = 0.991 *x* and *y* = 0.956 *x*, *R*^2^ = 0.801 and 0.711, and both slopes’ *P* < 0.001. The dashed red line marks the slope of 1.

**Figure 3.**
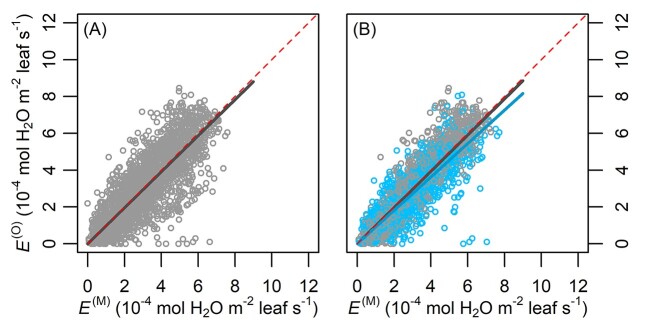
Overall performances of the reference models (RMs). The RMs are: (A) the base optimal stomata model (Eqs (4–9)) with *λ* directly estimated without soil-to-leaf conductance, and (B) a bridged model including soil-to-leaf conductance with a monotonic soil-to-root conductance (i.e. without accounting waterlogging effect). Dark gray and dark blue lines, respectively, denote the fittings using the full data (light gray circles) and those with shallow WTD ($<{\delta}^{\ast }-10\ \mathrm{cm}=37.98\ \mathrm{cm}$; Table 3; blue circles). The data set was not so separated for RM1 as it lacks the belowground structure. The dark gray fitted line in (A) is *y* = 0.979 *x* (*R*^2^ = 0.665), and the dark gray and dark blue fitted lines in (B) are, respectively, *y* = 0.984 *x* and *y* = 0.908 *x*, *R*^2^ = 0.776 and 0.697. All slopes’ *P* < 0.001.

**Figure 4.**
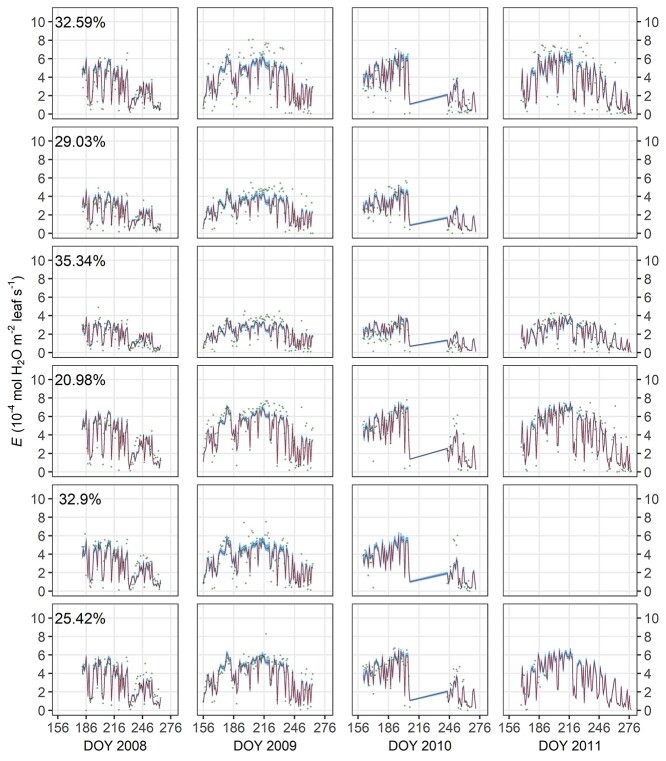
Comparison of observed transpiration rate *E*^(O)^ (green points) and simulated dynamics *E*^(M)^. Red line indicates the simulation using the parameters’ MAP estimates in each tree (Nos 1–6, from top to bottom). Blue shade shows the 95% credible interval of predictive uncertainty. The tree-specific normalized root-mean-square error (NRMSE, Eqs (20a) and (20b)) is labeled on the top left of the corresponding row. The data of trees Nos 2 and 5 were not available for 2011 as the signals were extremely noisy. DOY, day of year.

With the MAP estimates of the parameters (Table 3) and the full data, FM performed well in simulating transpiration rate, presenting a fitted slope of 0.991 (*R*^2^ = 0.801, Figure 2). In contrast, the fitted slope for RM1 (0.979) deviated more from 1, and its *R*^2^ was lower (0.665, Figure 3A). The performance of RM2 (slope = 0.984, *R*^2^ = 0.776) was close to that of FM when using the full data, but on shallow WTD ($<{\delta}^{\ast }-10\ \mathrm{cm}=37.98\ \mathrm{cm}$; Table 3) RM2’s overestimation of transpiration rate (fitted slope = 0.908, *R*^2^ = 0.697; Figure 3B) was larger than FM’s (fitted slope = 0.956, *R*^2^ = 0.711; Figures 2). All the fitted slopes were significant (*P* < 0.001). The tree-specific NRMSE of FM was low to moderate, ranging from 20.98 to 35.34% (Figure 4).

**Table 3:** MAP estimates and 95% credible intervals of the estimated parameters of the process model. Detailed information of the parameters (including their prior ranges) is shown in Table 2.

	MAP	2.5%	97.5%
*ξ*_*m*1_	0.084	0.071	0.145
*ξ*_*m*2_	0.100	0.071	0.148
*ξ*_*m*3_	0.097	0.074	0.147
*ξ*_*m*4_	0.146	0.108	0.149
*ξ*_*m*5_	0.103	0.072	0.148
*ξ*_*m*6_	0.106	0.074	0.148
*ξ_p_*	7.518	7.012	7.852
*η_m_*	0.056	0.050	0.094
*η_p_*	5.751	5.237	7.409
*δ* ^*^	46.9	36.3	49.9
*z*_0_	−3.800	−3.919	−3.801
*z*_1_	−0.783	−0.845	−0.780
*c*_1_	5.477 × 10^−2^	5.027 × 10^−2^	5.571 × 10^−2^
*c*_2_	5.295 × 10^−2^	5.015 × 10^−2^	5.656 × 10^−2^
*c*_3_	4.562 × 10^−2^	4.144 × 10^−2^	4.704 × 10^−2^
*c*_4_	6.994 × 10^−2^	6.766 × 10^−2^	6.997 × 10^−2^
*c*_5_	5.234 × 10^−2^	4.873 × 10^−2^	5.558 × 10^−2^
*c*_6_	5.329 × 10^−2^	4.990 × 10^−2^	5.513 × 10^−2^
*γ*	1.601 × 10^−3^	1.600 × 10^−3^	1.631 × 10^−3^

**Figure 5.**
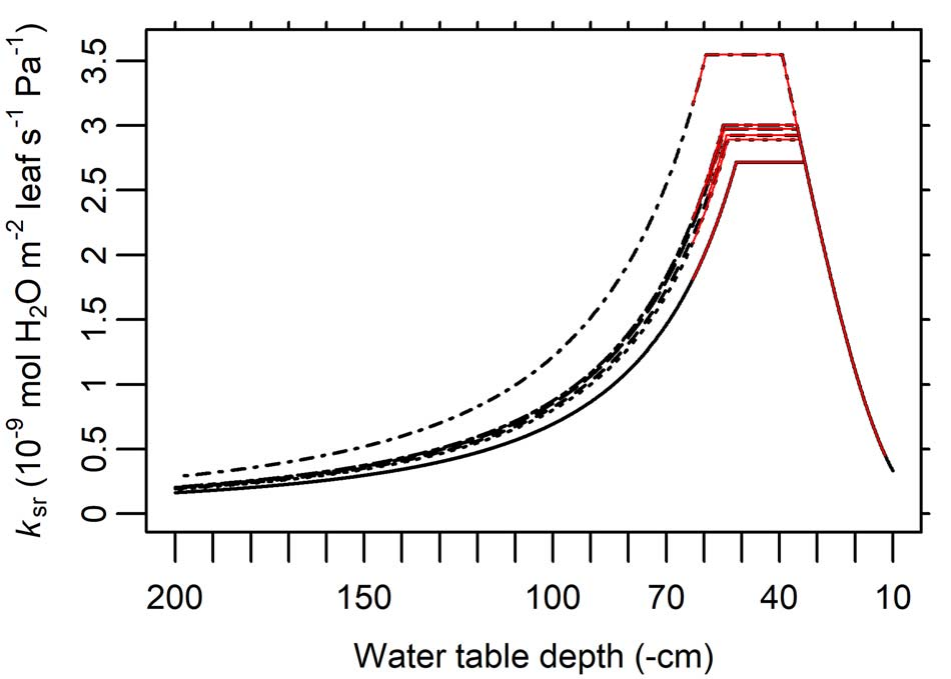
Simulated soil-to-root conductance (*k*_sr_, Eqs (13–16)) in relation to water table depth (WTD). Black lines are *k*_sr_ using the MAP estimates of the related parameters. From high to low at the maximum *k*_sr_ (‘plateau’) are trees Nos 4 (dot-dashed), 6 (long-dot-dashed), 5 (long-dashed), 2 (dashed), 3 (dotted) and 1 (solid line). Red solid segments demarcate the range of measured WTD.

**Figure 6.**
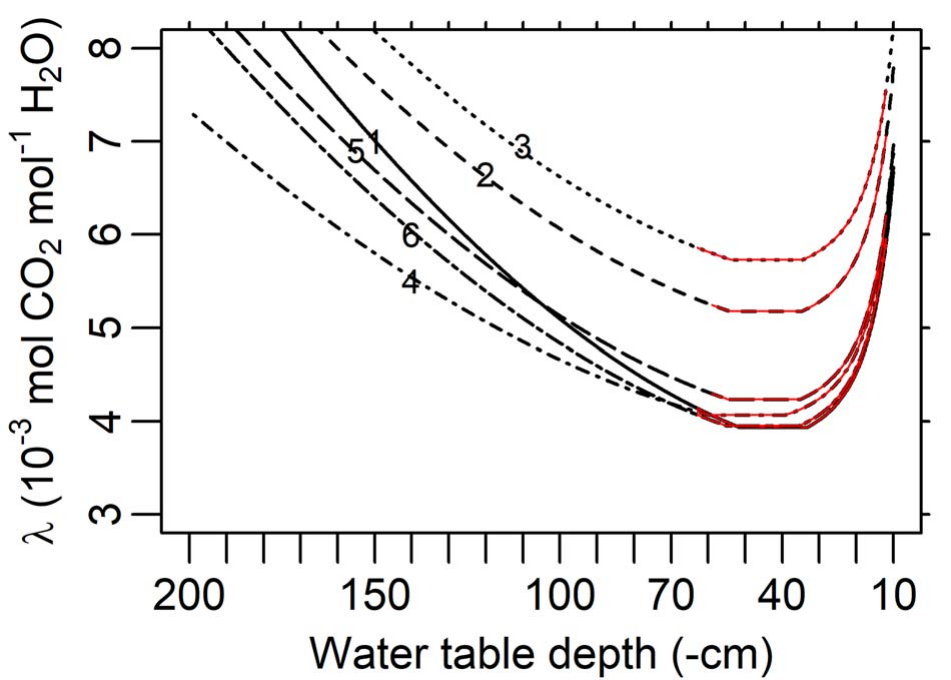
Simulated marginal water-use efficiency (*λ*, Eqs (10–16)) in relation to water table depth (WTD). Red solid segments demarcate the range of measured WTD. The parameterization of the simulation and the legend of lines (with tree numbers marked) are the same as in Figure 5.

The optimal WTD (δ^*^, i.e. the WTD through the stable phase of ${k}_{\mathrm{sr}}\big(\theta \big)$) was estimated to be 46.9 cm (Table 3), and thus, a decline in *k*_sr_ occurred from WTD = 33.4–39.3 cm (varying across trees) through shallower WTD levels (Figure 5). Correspondingly, the simulated maximum *k*_sr_ at the stable phase ranged between 2.72 × 10^−9^ and 3.55 × 10^−9^ mol H_2_O m^−2^ leaf s^−1^ Pa^−1^ (Figure 5). The simulated *λ* at δ^*^ ranged between 3.94 × 10^−3^ and 5.73 × 10^−3^ mol CO_2_ mol^−1^ H_2_O (Figure 6). The values of *λ* in all the trees increased sharply in waterlogged conditions (i.e. during the decrease of ${k}_{\mathrm{sr}}\big(\theta \big)$, Eq. (14), Figure 5), while a moderate rate of increase was found when WTD was deep. As a result, similar MWUE values were yielded at $\mathrm{WTD}=0.1\ \mathrm{m}$ and $\mathrm{WTD}\approx 1.5\ \mathrm{to}\ 2\ \mathrm{m}$ (varying across trees; Figure 6). The two taller trees (Nos 2 and 3, Table 1) had the highest *λ* values (Figure 6), but their lowest *k*_sr_ values did not differ notably from those of the other trees (Figure 5).

**Figure 7.**
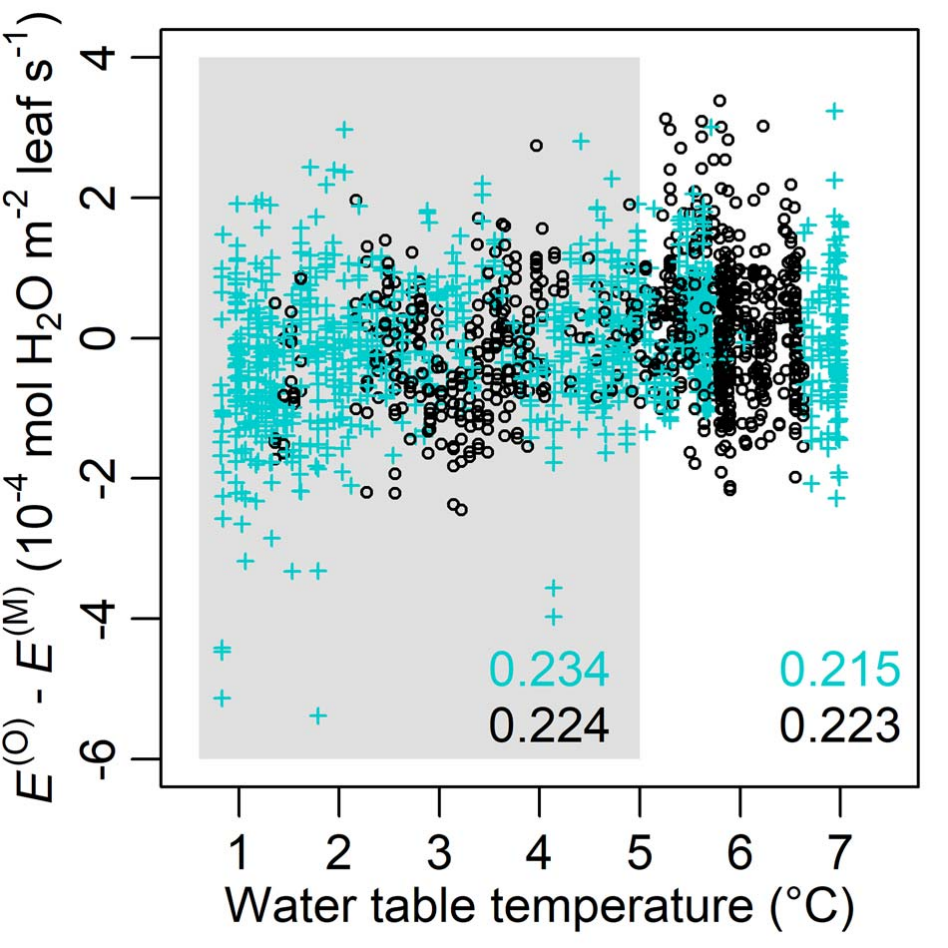
Model residual between observed and modeled transpiration rate $\big({E}^{\big(\mathrm{O}\big)}-{E}^{\big(\mathrm{M}\big)}\big)$ in relation to water table temperature (WTT). The gray box refers to $\mathrm{WTT}\le 5\ {}^{\circ}\mathrm{C}$. cyan crosses and black circles, respectively, indicate the data points within and outside the range of decreasing soil-to-root conductance (${k}_{\mathrm{sr}}^{-}\big(\theta \big)$, Eqs (14) and (16)). The color legend of data points (cyan and black) is the same as that of the Pearson correlation coefficient (*r*), which are labeled on the bottom right of the box (using the data with $\mathrm{WTT}\le 5\ {}^{\circ}\mathrm{C}$) and the panel (using the full data). All *P* < 0.001.

Of the full data, FM’s residual was significantly but not strongly correlated with WTT (*r* = 0.223), and the correlation was higher when $\mathrm{WTT}\le\! 5\ {}^{\circ}\mathrm{C}$ (*r* = 0.224, Figure 7). Under shallow WTD (< 36.9 cm) and $\mathrm{WTT}\le 5\ {}^{\circ}\mathrm{C}$, the residual and WTT had *r* = 0.234, but on the full WTT range the correlation was low (*r* = 0.215, Figure 7). In all cases, correlations were highly significant (*P* < 0.001).

## Discussion

A soil-to-leaf hydraulic conductance model was modified to reflect waterlogging effects, linked with stomatal optimization model, and parameterized with measured data from a drained peatland. Compared with the reference models (RM1 and RM2), the full model (FM) using the full data presented an *E*^(O)^:*E*^(M)^ fitted slope with the smallest difference from 1 and the highest *R*^2^. Particularly under shallow WTD, RM2 with monotonic ${k}_{\mathrm{sr}}\big(\theta \big)$ noticeably overestimated transpiration rate (${E}^{\big(\mathrm{O}\big)}=0.908\ {E}^{\big(\mathrm{M}\big)}$; Figure 3B), while FM yielded a closer estimation (${E}^{\big(\mathrm{O}\big)}=0.956\ {E}^{\big(\mathrm{M}\big)}$; Figure 2). FM was also accurate at individual tree level, presenting root-mean-square errors *c*. 1/5 to 1/3 of the observed means of respective trees (Figure 4). Thus, the hypothetical model structures of non-monotonic *k*_sr_ (Eqs (13–16), Figure 5) and $\lambda \big({k}_{\mathrm{sl}}\big)$ (Eq. (11), Figure 6) gained support from the data and the comparison with RMs without such designs.

### Model structure

The adverse effects of excess soil water on hydraulics and gas exchange in plants vary across taxa and soil types (Pezeshki and Chambers 1986, Oren et al. 2001, Ferner et al. 2012, Pearson et al. 2013), and we lack experimental observations of such effects under a gradient of SWC or WTD for continuous quantification. Thus, no precedent process-based models tackled this problem before the current attempt. Nevertheless, there has been consensus that hypoxia or anoxia is the key phenomenon that strongly alters the physiochemical properties and processes in soil related to plant physiology (Kreuzwieser and Rennenberg 2014). Furthermore, the consequent changes following hypoxia in the number and/or the status of aquaporins have been found to be generally the pivotal factor impacting soil-to-root conductance (Clarkson et al. 2000, Chaumont et al. 2005, Lambers et al. 2008, Johnson et al. 2014, Vandeleur et al. 2014, Domec et al. 2021). Particularly in flooding-sensitive pine species, it is likely that the decline of aquaporin activity under flooding is similar to that under drought (Domec et al. 2021). Therefore, we assumed the decreasing phase of soil-to-root conductance $\big({k}_{\mathrm{sr}}^{-}\big)$ to occur due to excess soil water, which is related to the oxygen availability controlled by the available soil air space and diffusion (Eq. (14)). Despite the algebraically simplistic design, this hypothetical structure successfully simulated the transpiration rate of Scots pine trees in a drained peatland over four growing seasons (Figures 2 and 4). Also, the performance was better than that of RMs representing (i) stomatal behavior influenced by *D*, *I* and *T*_l_ alone or (ii) with monotonic correlation between SWC and *k*_sr_ as in previous studies (Duursma et al. 2008, Nikinmaa et al. 2013, Hölttä et al. 2017, Figure 3). With regard to these results, we conclude that the new model corroborates the prior findings on hypoxia and aquaporins in the perspective of soil–tree–atmosphere continuum and provides new quantitative insights into the water uptake and use of trees under waterlogged conditions in drained peatland.

### Soil-to-root conductance, water use and water table depth

The maximum simulated *k*_sr_ (Figure 5) did not show significant negative correlation with tree height (correlation coefficient *r* < 0 but *P* > 0.5), in contrast with the belowground conductance significantly declining with tree height in previous observations (Martínez-Vilalta et al. 2007*a*). This distinction might be related to the different modeling method and more homogeneous sample trees in the current study. Instead of the simple one-membrane two-compartment (above- and belowground) model in the reference study (Martínez-Vilalta et al. 2007*a*), the current model employed a root structure module with more details, which was compacted into the tree-specific parameter *ξ_mi_* (Eq. (13)). Ecophysiologically, ${\xi}_m={R}_L\frac{2\pi }{\ln \big({r}_{\mathrm{cyl}}/{r}_{\mathrm{root}}\big)}{K}_{\mathrm{sat}}$, where *R_L_* is root length to all-sided leaf area ratio (*m* root m^−2^ leaf), ${r}_{\mathrm{cyl}}/{r}_{\mathrm{root}}$ the ratio of hydraulically active rhizosphere cylinder to average fine root radii (both in *m*), and *K*_sat_ the saturated conductivity of the soil (mol H_2_O m^−1^ s^−1^ Pa^−1^) (Nikinmaa et al. 2013). As the sample trees grew closely in one stand with narrow height and age spans, it is reasonable to assume that none of *K*_sat_, *R*_L_ or ${r}_{\mathrm{cyl}}/{r}_{\mathrm{root}}$ varied significantly across the trees. In uneven-aged stands, ${r}_{\mathrm{cyl}}/{r}_{\mathrm{root}}$ may particularly be positively correlated with tree age/height as a result of greater belowground carbon allocation of old/taller trees, thereby presenting *k*_sr_ declining with tree age/height as well. However, new observations are required to verify this hypothesis.

As *k*_sr_ declines in waterlogged conditions, *λ* steeply increases, and its values of the two taller trees (Nos 2 and 3, Table 1) are distinct from those of the other four (Figure 6). This difference, more significant than in *k*_sr_, was partially caused by a higher hydraulic resistance between roots and leaves (i.e. lower *k*_rl_, Eqs (10) and (12)), represented by their lower ${J}_{\mathrm{max}}^{\big(\mathrm{O}\big)}$ (Table 1), *E*^(O)^ and *E*^(M)^ (Figure 4). These results are in accordance with the hypothesis on hydraulic resistance being the main limiting factor of trees’ assimilation and height growth, namely, the hydraulic limitation hypothesis (HLH). Widely supported by modeling and observations (Hubbard et al. 1999, West et al. 1999, McDowell et al. 2002*b*, Koch et al. 2004, Martínez-Vilalta et al. 2007*b*, Drake et al. 2010, Olson et al. 2014), HLH states that increasing hydraulic resistance against water transport from soil to leaves is the key constraint on the transpiration and photosynthesis of old/tall trees (Ryan and Yoder 1997, Ryan et al. 2006). The tests on this hypothesis have always been on very tall trees (Koch et al. 2004) and/or under drought (Sevanto et al. 2014), while the current study suggests that lower transpiration rate and higher MWUE related to lower soil-to-leaf conductance are noticeable also in short (10–15 m) trees with excess soil water. However, nutrient cycles might have affected the trees’ growth too regarding the slowed decomposition under subarctic conditions. Further research is required to clarify this issue.

### Residual and water table temperature

Suggested by the correlation between FM’s residual (*ε*) and WTT (*r* = 0.228 using the full data and 0.237 in shallow WTD; Figure 7), the model slightly overestimated transpiration rate when WTT was close to 0 °C, but the residual diminished as WTT increased to 5 °C. This correlation may be explained by (i) lower diffusion coefficient of oxygen in colder soils (Chapman-Enskog theory) and (ii) higher plasma membrane resistance in roots to water at low temperature (Lambers et al. 2008), and the main reason behind the latter is likely the reduced activity of aquaporins (Javot and Maurel 2002, Murai-Hatano et al. 2008, Ionenko et al. 2010; but see section *Caveats* for the potential impacts of seasonality absent from the current structure). Thus, including WTT as an input variable in the model may potentially improve the model’s performance on soil-to-root hydraulic conductance. However, on the whole-tree scale, the effects of belowground temperature barely over the freezing point on hydraulic conductance or transpiration are yet unclear (but see Running and Reid (1980), Mellander et al. (2004) and Lintunen et al. (2020) for the influences of wider ranges of soil temperature). Also, the current ${k}_{\mathrm{sr}}^{-}\big(\theta \big)$ does not yet explain detailed ecophysiological processes associated with waterlogging or WTT (see the next section for these limitations of the model). Therefore, more observations and process-based studies are necessary before the implementation of WTT.

### Caveats

There were several structural or parametric assumptions in the current modeling to overcome the difficulties in measurements and/or computation. Firstly, due to lack of previous observations over a gradient of shallow WTD or high SWC, ${k}_{\mathrm{sr}}^{-}\big(\theta \big)$ (Eq. (14)) was modeled using simplified coefficients *η_m_* and *η_p_*, of which the ecophysiological interpretation is not straightforward, unlike that of *ξ_m_* and *ξ_p_* for ${k}_{\mathrm{sr}}^{+}\big(\theta \big)$ (Eq. (13); Duursma et al. 2008, Nikinmaa et al. 2013). Despite the formula’s superficial resemblance to the model of oxygen diffusion in soil (Moldrup et al. 1997), *η_m_* and *η_p_* lump together a variety of factors, e.g. aquaporin dynamics, respiration-related enzymes dynamics, and soil gas composition. Therefore, this part of the model provides only a heuristic quantification, and more mechanistic understanding is needed for modeling these processes in the future.

Secondly, the parameters not directly related to the focal properties and processes (MWUE, hydraulic conductance and waterlogging) were kept constant through the study. Of the estimated parameters, only *ξ_m_* and *c* were tree specific. The constant or non-tree-specific parameters may have introduced errors into the results due to site- or tree-level variances in ecophysiological properties, e.g. lowest water potential at tree top (${\Psi}_{\mathrm{lmin}}$ in Eq. (12)), saturation of irradiance reaction (*γ* in Eq. (4)) and leaf-to-sapwood area ratio (*ρ* in Eqs (12) and (18)). These errors, in addition to the novel model structures, may have contributed to the ranges of *k*_sr_ and/or *λ* results different from those of previous modeling studies (Mäkelä et al. 2004, Duursma et al. 2008, Liu et al. 2020). Particularly, leaf-to-sapwood area ratio in Scots pine has been found decreasing with increasing tree height (Vanninen et al. 1996, McDowell et al. 2002*a*). Therefore, assuming the ratio constant (*ρ* = 2500 m^2^ leaf m^−2^ sapwood; Table S1 available as Supplementary data at *Tree Physiology* Online) should have induced an error in converting sap flow density to leaf-area-specific transpiration rate (Eq. (18)), albeit the error should be minor due to the limited difference among the heights of the sample trees (Table 1).

Moreover, no processes except *k*_sr_ were assumed directly correlated with SWC or WTD, while, for instance, the maximum photosynthetic rate (reflected by parameter *γ* in the current model) may be negatively correlated with SWC under waterlogged conditions (Sojka 1992, Carter et al. 2006). The trees might have acclimated to waterlogging or flooding conditions so that their impacts on hydraulic conductance and MWUE might have been mediated to some extent. Such an acclimation may be similar to the flooding-tolerance observed in Scots pine seedlings grown on peat (Pearson et al. 2013). Therefore, cautious scrutiny on ecophysiological processes and phenomena is necessary when the current modeling method is applied to the trees in different landscapes or soil conditions.

A number of measurement points scatter outside the 95% credible interval of predictive error (Figure 4), suggesting considerable measurement error additional to the aforementioned structural or parametric limits. Particularly, the measurement error in WTD and *J* should be emphasized. In drained peatland, the soil water distribution may be highly correlated with the distance between the points of interest and the nearest draining ditch (Laurén et al. 2021). Thus, the automatically recorded WTD at the approximate center of the site may have induced error when used as input data supplementary to the tree-specific manual measurement. The error in the thermal dissipation method of measuring sap flow density has been widely discussed, such as on the sensitivity to direct sunshine and the thermal sensitivity of the device (Köstner et al. 1998, Hölttä et al. 2015). These issues in WTD and sap flow density data may have caused the heteroscedasticity in the data model (Eq. (19)), which was ubiquitous across trees but in different correlations with *E*^(M)^. Considering the limitations in modeling methodology and data, we expect to improve the model with measurements of more environmental factors (e.g. oxygen content and/or diffusion rate in soil) and by more accurate measurement methods.

## Conclusion

Our semi-mechanistic model of soil to leaf hydraulic continuum performed well on data collected from six Scots pine trees in a drained peatland with seasonal waterlogging. It performed better than did the original optimal stomata model alone and the other reference model (RM2) structured similarly but without the waterlogging module. Particularly, the full new model’s simulated transpiration rate of the sample trees had a much less deviation from the observations than the results of RM2. In the future, the model could be improved on providing more mechanistic expressions of related processes, e.g. oxygen diffusion in soil, roots’ water uptake and respiration. Additionally, continuous measurements of more variables, e.g. respiration rate, soil oxygen concentration, and water potentials at canopy and soil, should improve the model’s performance and applicability.

## Supplementary Material

SI_tpac037

## Data and materials availability

The raw and processed data and the R code for model parameterization are available upon request to the authors.

## References

[ref1] Alaoui-Sossé B , Gérard B, Binet P, Toussaint M-L, Badot P-M (2005) Influence of flooding on growth nitrogen availability in soil and nitrate reduction of young oak seedlings (*Quercus robur* L). Ann For Sci 62:593–600.

[ref2] Bell DM , Ward EJ, Ach O, Oren R, Flikkema PG, Clark JS (2015) A state-space modeling approach to estimating canopy conductance and associated uncertainties from sap flux density data. Tree Physiol 35:792–802. 26063709 10.1093/treephys/tpv041

[ref3] Campbell GS , Norman J (1998) An introduction to environmental biophysics. Springer Science and Business Media, New York, NY.

[ref4] Carter JL , Colmer TD, Veneklaas EJ (2006) Variable tolerance of wetland tree species to combined salinity and waterlogging is related to regulation of ion uptake and production of organic solutes. New Phytol 169:123–134. 16390424 10.1111/j.1469-8137.2005.01552.x

[ref5] Chaumont F , Moshelion M, Daniels MJ (2005) Regulation of plant aquaporin activity. Biol Cell 97:749–764. 16171457 10.1042/BC20040133

[ref6] Christensen JH , Christensen OB (2003) Severe summertime flooding in Europe. Nature 421:805–806. 12594501 10.1038/421805a

[ref7] Christmann A , Grill E, Huang J (2013) Hydraulic signals in long-distance signaling. Curr Opin Plant Biol 16:293–300. 23545219 10.1016/j.pbi.2013.02.011

[ref8] Clarkson DT , Carvajal M, Henzler T, Waterhouse RN, Smyth AJ, Cooke DT, Steudle E (2000) Root hydraulic conductance: diurnal aquaporin expression and the effects of nutrient stress. J Exp Bot 51:61–70. 10938796

[ref9] Cowan IR (1977) Stomatal behaviour and environment. Adv Bot Res 4:117–228.

[ref10] Cowan IR , Farquhar GD (1977) Stomatal function in relation to leaf metabolism and environment. In: Jennings DH (ed) Integration of activity in the higher plant. Cambridge University Press Cambridge, UK, pp. 471–505. 756635

[ref11] Dewar R , Mauranen A, Mäkelä A, Hölttä T, Medlyn B, Vesala T (2018) New insights into the covariation of stomatal mesophyll and hydraulic conductances from optimization models incorporating nonstomatal limitations to photosynthesis. New Phytol 217:571–585. 29086921 10.1111/nph.14848

[ref12] Dewar RC (2002) The Ball-Berry-Leuning and Tardieu-Davies stomatal models: synthesis and extension within a spatially aggregated picture of guard cell function. Plant Cell Environ 25:1383–1398.

[ref13] Domec J-C , King JS, Carmichael MJ, Overby AT, Wortemann R, Smith WK, Miao G-F, Noormets A, Johnson DM (2021) Aquaporins and not changes in root structure provide new insights into physiological responses to drought flooding and salinity. J Exp Bot 72:4489–4501. 33677600 10.1093/jxb/erab100

[ref14] Drake JE , Raetz LM, Davis SC, Delucia EH (2010) Hydraulic limitation not declining nitrogen availability causes the age-related photosynthetic decline in loblolly pine (*Pinus taeda* L). Plant Cell Environ 33:1756–1766. 20545880 10.1111/j.1365-3040.2010.02180.x

[ref15] Duursma RA , Kolari P, Perämäki M, et al. (2008) Predicting the decline in daily maximum transpiration rate of two pine stands during drought based on constant minimum leaf water potential and plant hydraulic conductance. Tree Physiol 28:265–276. 18055437 10.1093/treephys/28.2.265

[ref16] Ferner E , Rennenberg H, Kreuzwieser J (2012) Effect of flooding on C metabolism of flood-tolerant (*Quercus robur*) and non-tolerant (*Fagus sylvatica*) tree species. Tree Physiol 32:135–145. 22367762 10.1093/treephys/tps009

[ref17] Ferry B , Morneau F, Bontemps J-D, Blanc L, Freycon V (2010) Higher treefall rates on slopes and waterlogged soils result in lower stand biomass and productivity in a tropical rain forest. J Ecol 98:106–116.

[ref18] Frei C , Schöll R, Fukutome S, Schmidli J, Vidale PL (2006) Future change of precipitation extremes in Europe: intercomparison of scenarios from regional climate models. J Geophys Res Atmos 111:D06105.

[ref19] Gelman A , Carlin JB, Stern HS, Dunson DB, Vehtari A, Rubin DB (2013) Bayesian data analysis, 3rd edn. CRC Press, Boca Raton, FL.

[ref20] Granier A (1987) Evaluation of transpiration in a Douglas-fir stand by means of sap flow measurements. Tree Physiol 3:309–320. 14975915 10.1093/treephys/3.4.309

[ref21] Hari P , Mäkelä A (2003) Annual pattern of photosynthesis in scots pine in the boreal zone. Tree Physiol 23:145–155. 12566265 10.1093/treephys/23.3.145

[ref22] Hari P , Mäkelä A, Korpilahti E, Holmberg M (1986) Optimal control of gas exchange. Tree Physiol 2:169–175. 14975851 10.1093/treephys/2.1-2-3.169

[ref23] Hartig F , Minunno F, Paul S (2019) Bayesian tools: general-purpose MCMC and SMC samplers and tools for Bayesian statistics. R package version 0.1.6. https://CRANR-project.org/package=BayesianTools.

[ref24] Hökkä H , Laurén A, Stenberg L, Launiainen S, Leppä K, Nieminen M (2021) Defining guidelines for ditch depth in drained scots pine dominated peatland forests. Silva Fenn 55:10494.

[ref25] Hölttä T , Linkosalo T, Riikonen A, Sevanto S, Nikinmaa E (2015) An analysis of Granier sap flow method its sensitivity to heat storage and a new approach to improve its time dynamics. Agric For Meteorol 211:2–12.

[ref26] Hölttä T , Lintunen A, Chan T, Mäkelä A, Nikinmaa E (2017) A steady-state stomatal model of balanced leaf gas exchange hydraulics and maximal source-sink flux. Tree Physiol 37:851–868. 28338800 10.1093/treephys/tpx011

[ref27] Hubbard RM , Bond BJ, Ryan MG (1999) Evidence that hydraulic conductance limits photosynthesis in old *Pinus ponderosa* trees. Tree Physiol 19:165–172. 12651579 10.1093/treephys/19.3.165

[ref28] Ionenko IF , Anisimov AV, Dautova NR (2010) Effect of temperature on water transport through aquaporins. Biol Plant 54:488–494.

[ref29] IPCC (2021) Climate change 2021: the physical science basis. Contributions of working groups I to the sixth assessment report of the Intergovernmental Panel on Climate Change. Cambridge University Press, UK (in press). https://www.ipcc.ch/report/ar6/wg1/downloads/report/IPCC_AR6_WGI_Full_Report.pdf (13 September 2021, date last accessed).

[ref30] Jackson MB (2002) Long-distance signalling from roots to shoots assessed: the flooding story. J Exp Bot 53:175–181. 11807120 10.1093/jexbot/53.367.175

[ref31] Javot H , Maurel C (2002) The role of aquaporins in root water uptake. Ann Bot 90:301–313. 12234142 10.1093/aob/mcf199 PMC4240399

[ref32] Johnson DM , Sherrard ME, Domec J-C, Jackson RB (2014) Role of aquaporin activity in regulating deep and shallow root hydraulic conductance during extreme drought. Trees 28:1323–1331.

[ref33] Katul G , Manzoni S, Palmroth S, Oren R (2010) A stomatal optimization theory to describe the effects of atmospheric CO_2_ on leaf photosynthesis and transpiration. Ann Bot 105:431–442. 19995810 10.1093/aob/mcp292 PMC2826246

[ref34] Koch GW , Sillett SC, Jennings GM, Davis SD (2004) The limits to tree height. Nature 428:851–854. 15103376 10.1038/nature02417

[ref35] Kolari P , Lappalainen HK, Hänninen H, Hari P (2007) Relationship between temperature and the seasonal course of photosynthesis in scots pine at northern timberline and in southern boreal zone. Tellus B Chem Phys Meteorol 59:542–552.

[ref36] Köstner B , Granier A, Cermák J (1998) Sapflow measurements in forest stands: methods and uncertainties. Ann Sci For 55:13–27.

[ref37] Kreuzwieser J , Rennenberg H (2014) Molecular and physiological responses of trees to waterlogging stress. Plant Cell Environ 37:2245–2259. 24611781 10.1111/pce.12310

[ref38] Lambers H , Chapin FS III, Pons TL (2008) Plant physiological ecology, 2nd edn. Springer Science and Business Media, New York, NY.

[ref39] Laurén A , Palviainen M, Launiainen S, Leppä K, Stenberg L, Urzainki I, Nieminen M, Laiho R, Hökkä H (2021) Drainage and stand growth response in peatland forests—description testing and application of mechanistic peatland simulator SUSI. Forests 12:293.

[ref40] Leppä K , Hökkä H, Laiho R, Launiainen S, Lehtonen A, Mäkipää R, Peltoniemi M, Saarinen M, Sarkkola S, Nieminen M (2020) Selection cuttings as a tool to control water table level in boreal drained peatland forests. Front Earth Sci 8:428.

[ref41] Lin Y-S , Medlyn BE, Duursma RA et al. (2015) Optimal stomatal behaviour around the world. Nat Clim Chang 5:459–464.

[ref42] Lintunen A , Paljakka T, Salmon Y, Dewar R, Riikonen A, Hölttä T (2020) The influence of soil temperature and water content on belowground hydraulic conductance and leaf gas exchange in mature trees of three boreal species. Plant Cell Environ 43:532–547. 31873942 10.1111/pce.13709

[ref43] Liu C , Hölttä T, Tian X-L, Berninger F, Mäkelä A (2020) Weaker light response lower stomatal conductance and structural changes in old boreal conifers implied by a Bayesian hierarchical model. Front Plant Sci 11:579319. 33240299 10.3389/fpls.2020.579319 PMC7677260

[ref44] Lu P , Urban L, Zhao P (2004) Granier's thermal dissipation probe (TDP) method for measuring sap flow in trees: theory and practice. Acta Bot Sin 46:631–646.

[ref45] Lu Y , Duursma RA, Farrior CE, Medlyn BE, Feng X (2020) Optimal stomatal drought response shaped by competition for water and hydraulic risk can explain plant trait covariation. New Phytol 225:1206–1217. 31538667 10.1111/nph.16207

[ref46] Mäkelä A , Berninger F, Hari P (1996) Optimal control of gas exchange during drought: theoretical analysis. Ann Bot 77:461–468.

[ref47] Mäkelä A , Hari P, Berninger F, Hänninen H, Nikinmaa E (2004) Acclimation of photosynthetic capacity in scots pine to the annual cycle of temperature. Tree Physiol 24:369–376. 14757576 10.1093/treephys/24.4.369

[ref48] Mäkelä A , Pulkkinen M, Kolari P, et al. (2008) Developing an empirical model of stand GPP with the LUE approach: analysis of eddy covariance data at five contrasting conifer sites in Europe. Glob Chang Biol 14:92–108.

[ref49] Manzoni S , Vico G, Palmroth S, Porporato A, Katul G (2013) Optimization of stomatal conductance for maximum carbon gain under dynamic soil moisture. Adv Water Resour 62:90–105.

[ref51] Martínez-Vilalta J , Korakaki E, Vanderklein D, Mencuccini M (2007 *a*) Below-ground hydraulic conductance is a function of environmental conditions and tree size in scots pine. Funct Ecol 21:1072–1083.

[ref52] Martínez-Vilalta J , Vanderklein D, Mencuccini M (2007 *b*) Tree height and age-related decline in growth in scots pine (*Pinus sylvestris* L). Oecologia 150:529–544. 16983553 10.1007/s00442-006-0552-7

[ref50] Martínez-Vilalta J , Cochard H, Mencuccini M et al. (2009) Hydraulic adjustment of scots pine across Europe. New Phytol 184:353–364. 19674333 10.1111/j.1469-8137.2009.02954.x

[ref53] McDowell N , Barnard H, Bond B et al. (2002 *a*) The relationship between tree height and leaf area: sapwood area ratio. Oecologia 132:12–20. 28547290 10.1007/s00442-002-0904-x

[ref54] McDowell NG , Phillips N, Lunch C, Bond BJ, Ryan MG (2002 *b*) An investigation of hydraulic limitation and compensation in large old Douglas-fir trees. Tree Physiol 22:763–774. 12184980 10.1093/treephys/22.11.763

[ref55] Medlyn BE , Duursma RA, Eamus D, Ellsworth DS, Prentice IC, Barton CV, Wingate L (2011) Reconciling the optimal and empirical approaches to modelling stomatal conductance. Glob Chang Biol 17:2134–2144.

[ref56] Mellander PE , Bishop K, Lundmark T (2004) The influence of soil temperature on transpiration: a plot scale manipulation in a young scots pine stand. For Ecol Manage 195:15–28.

[ref57] Moldrup P , Kruse CW, Rolston DE, Yamaguchi T (1996) Modeling diffusion and reaction in soils: III predicting gas diffusivity from the Campbell soil-water retention model. Soil Sci 161:366–375.

[ref58] Moldrup P , Olesen T, Rolston DE, Yamaguchi T (1997) Modeling diffusion and reaction in soils: VII predicting gas and ion diffusivity in undisturbed and sieved soils. Soil Sci 162:632–640.

[ref59] Murai-Hatano M , Kuwagata T, Sakurai J, et al. (2008) Effect of low root temperature on hydraulic conductivity of rice plants and the possible role of aquaporins. Plant Cell Physiol 49:1294–1305. 18676378 10.1093/pcp/pcn104

[ref60] Nikinmaa E , Hölttä T, Hari P, Kolari P, Mäkelä A, Sevanto S, Vesala T (2013) Assimilate transport in phloem sets conditions for leaf gas exchange. Plant Cell Environ 36:655–669. 22934921 10.1111/pce.12004

[ref61] Oishi AC , Hawthorne DA, Oren R (2016) Baseliner: an open-source, interactive tool for processing sap flux data from thermal dissipation probes. SoftwareX 5:139–143.

[ref63] Olson ME , Anfodillo T, Rosell JA, Petit G, Crivellaro A, Isnard S, León-Gómez C, Alvarado-Cárdenas LO, Castorena M (2014) Universal hydraulics of the flowering plants: vessel diameter scales with stem length across angiosperm lineages habits and climates. Ecol Lett 17:988–997. 24847972 10.1111/ele.12302

[ref64] Oren R , Sperry JS, Ewers BE, Pataki DE, Phillips N, Megonigal JP (2001) Sensitivity of mean canopy stomatal conductance to vapor pressure deficit in a flooded *Taxodium distichum* L forest: hydraulic and non-hydraulic effects. Oecologia 126:21–29. 28547434 10.1007/s004420000497

[ref65] Päivänen J (1973) Hydraulic conductivity and water retention in peat soils. Suomen Metsätieteellinen Seura, Helsinki, Finland.

[ref66] Palmer TN , Räisänen J (2002) Quantifying the risk of extreme seasonal precipitation events in a changing climate. Nature 415:512–514. 11823856 10.1038/415512a

[ref67] Parent C , Capelli N, Berger A, Crèvecoeur M, Dat JF (2008) An overview of plant responses to soil waterlogging. Plant Stress 2:20–27.

[ref68] Parolin P (2001) Morphological and physiological adjustments to waterlogging and drought in seedlings of Amazonian floodplain trees. Oecologia 128:326–335. 24549901 10.1007/s004420100660

[ref69] Pearson M , Saarinen M, Nummelin L, Heiskanen J, Roitto M, Sarjala T, Laine J (2013) Tolerance of peat-grown scots pine seedlings to waterlogging and drought: morphological physiological and metabolic responses to stress. For Ecol Manage 307:43–53.

[ref70] Pezeshki SR , Chambers JL (1986) Variation in flood-induced stomatal and photosynthetic responses of three bottomland tree species. For Sci 32:914–923.

[ref71] Pezeshki SR , Pardue JH, DeLaune RD (1996) Leaf gas exchange and growth of flood-tolerant and flood-sensitive tree species under low soil redox conditions. Tree Physiol 16:453–458. 14871732 10.1093/treephys/16.4.453

[ref72] Poyatos R , Aguadé D, Martínez-Vilalta J (2018) Below-ground hydraulic constraints during drought-induced decline in scots pine. Ann For Sci 75:1–14.

[ref73] Prentice IC , Dong N, Gleason SM, Maire V, Wright IJ (2014) Balancing the costs of carbon gain and water transport: testing a new theoretical framework for plant functional ecology. Ecol Lett 17:82–91. 24215231 10.1111/ele.12211

[ref74] R Development Core Team . (2020) R: a language and environment for statistical computing. R Foundation for Statistical Computing Vienna, Austria. https://www.R-project.org/.

[ref75] Repo T , Domisch T, Kilpeläinen J, Piirainen S, Silvennoinen R, Lehto T (2020) Dynamics of fine-root production and mortality of scots pine in waterlogged peat soil during the growing season. Can J For Res 50:510–518.

[ref76] Running SW , Reid CP (1980) Soil temperature influences on root resistance of *Pinus contorta* seedlings. Plant Physiol 65:635–640. 16661254 10.1104/pp.65.4.635 PMC440398

[ref78] Ryan MG , Yoder BJ (1997) Hydraulic limits to tree height and tree growth. Bioscience 47:235–242.

[ref77] Ryan MG , Phillips N, Bond BJ (2006) The hydraulic limitation hypothesis revisited. Plant Cell Environ 29:367–381. 17080592 10.1111/j.1365-3040.2005.01478.x

[ref79] Schiestl-Aalto P , Kulmala L, Mäkinen H, Nikinmaa E, Mäkelä A (2015) CASSIA—a dynamic model for predicting intra-annual sink demand and interannual growth variation in scots pine. New Phytol 206:647–659. 25616175 10.1111/nph.13275

[ref80] Sevanto S , McDowell NG, Dickman LT, Pangle R, Pockman WT (2014) How do trees die? A test of the hydraulic failure and carbon starvation hypotheses. Plant Cell Environ 37:153–161. 23730972 10.1111/pce.12141 PMC4280888

[ref81] Sojka RE (1992) Stomatal closure in oxygen-stressed plants. Soil Sci 154:269–280.

[ref82] Stenberg L , Haahti K, Hökkä H, Launiainen S, Nieminen M, Laurén A, Koivusalo H (2018) Hydrology of drained peatland forest: numerical experiment on the role of tree stand heterogeneity and management. Forests 9:645.

[ref83] Suni T , Berninger F, Vesala T et al. (2003) Air temperature triggers the recovery of evergreen boreal forest photosynthesis in spring. Glob Chang Biol 9:1410–1426.

[ref84] Vandeleur RK , Sullivan W, Athman A, Jordans C, Gilliham M, Kaiser BN, Tyerman SD (2014) Rapid shoot-to-root signalling regulates root hydraulic conductance via aquaporins. Plant Cell Environ 37:520–538. 23926961 10.1111/pce.12175

[ref85] Vanninen P , Ylitalo H, Sievänen R, Mäkelä A (1996) Effects of age and site quality on the distribution of biomass in scots pine (*Pinus sylvestris* L). Trees 10:231–238.

[ref86] Vasander H , Laine J (2008) Site type classification on drained peatlands. In: Korhonen R, Korpela L, Sarkkola S (eds) Finland-Fenland: research and sustainable utilisation of mires and peat. Finnish Peatland Society and Maahenki Ltd., Helsinki, Finland, pp. 146–151.

[ref87] Vrugt JA , Ter Braak CJF, Diks CGH, Robinson BA, Hyman JM, Higdon D (2009) Accelerating Markov chain Monte Carlo simulation by differential evolution with self-adaptive randomized subspace sampling. Int J Nonlinear Sci Numer Simul 10:273–290.

[ref88] West GB , Brown JH, Enquist BJ (1999) A general model for the structure and allometry of plant vascular systems. Nature 400:664–667.

[ref89] Wolf A , Anderegg WR, Pacala SW (2016) Optimal stomatal behavior with competition for water and risk of hydraulic impairment. Proc Natl Acad Sci USA 113:E7222–E7230. 27799540 10.1073/pnas.1615144113 PMC5135368

