# Supplementary material for: A model bridging waterlogging, stomatal behavior and water use in trees in drained peatland
Source: Tree Physiol. 2022 Apr 6;42(9):1736–49. doi: 10.1093/treephys/tpac037 (PMC9460983; doi:10.1093/treephys/tpac037)
Supplement: SI_tpac037 [file si_tpac037.docx]

**SUPPLEMENTARY INFORMATION**

**Table S1. List of symbols**

| Symbol | Type † | Meaning | Value and/or unit  (if applicable) | Source of value (if applicable) |
| --- | --- | --- | --- | --- |
| *c* | Pe | Slope of acclimation of foliage to temperature | m^3^ (mol C°)^-1^ |  |
| *C*_a_ | C | CO_2_ concentration in the atmosphere | 1.669 × 10^-2^ mol m^-3^ | Finnish Meteorological Institute |
| *D* | V | Vapour (pressure) deficit | mol m^-3^ |  |
| *E*^(M)^, *E*^(O)^ | V | Modelled and observed transpiration rate, respectively | mol H_2_O m^-2^ leaf s^-1^ |  |
| *g*_σ_ | V | Stomatal conductance | m s^-1^ |  |
| *h*_r_ | Vo | Relative humidity | (%) |  |
| *i* | S | Ordinal number of tree |  |  |
| *I* | Vo | Photosynthetic photon flux rate (PPFD) | mol m^-2^ leaf s^-1^ |  |
| *j* | S | Index of data point in time series |  |  |
| *J* | Vo | (Measured) Sap flow density | mol H_2_O m^-2^ sapwood  s^-1^ |  |
| *k*_0_ | C | Base-case soil-to-leaf conductance | 2.22 × 10^-8^ mol H_2_O m^-2^ leaf s^-1^ Pa^-1^ | Nikinmaa et al (2013); Hölttä et al (2017) |
| *k*_sl_, *k*_sr_, *k*_rl_ | V | Conductances for water between soil and leaf, soil and root, and root and leaf, respectively | mol H_2_O m^-2^ leaf s^-1^ Pa^-1^ |  |
| *Q*_10_ | C | Relative increase of *R* per 10 °C | 2.3 | Mäkelä et al (2004) |
| *R* | V | Respiration rate | mol CO_2_ m^-2^ leaf s^-1^ |  |
| *R*_0_ | C | Value of *R* at 0 °C | 9.1 × 10^-8^ mol CO_2_ m^-2^ leaf s^-1^ | Mäkelä et al (2004) |
| *S* | V | Photosynthetic acclimation of foliage to temperature above *S*_0_ | °C |  |
| *S*_0_ | C | Threshold in the photosynthetic acclimation of foliage to temperature | -4.5 °C | Mäkelä et al (2004) |
| *T* | Vo | Air temperature | °C |  |
| *T*_l_ | V | Leaf temperature | °C |  |
| *z*_0_, *z*_1_ | Pe | Coefficients in the log-log linear correlation between *λ* and ${k_{\mathrm{sr}}}/{k_{0}}$ |  |  |
| *α* | Pe | Intercept of the heteroscedasticity as semi-log linear correlation with *E*^(M)^ for trees No. 4 & 6 |  |  |
| *β* | Pe | Slope of the heteroscedasticity as simple (trees No. 1, 2, 3 & 5) or semi-log (No. 4 & 6) linear correlation with *E*^(M)^ |  |  |
| *γ* | Pe | Saturation level of irradiance response curve | m s^-1^ |  |
| *δ* | Vo | Water table depth | m |  |
| *δ** | Pe | Optimal water table depth | m |  |
| ΔΨ_max_ | C | Maximum water potential difference between foliage and soil | -2 × 10^6^ Pa | Martínez-Vilalta et al (2009) |
| *η*_m_ | Pe | Multiplier coefficient in the power function $k_{\mathrm{sr}}\left( \theta\right)$ when decreasing | mol m^-2^ s^-1^ Pa^-1^ |  |
| *η*_p_ | Pe | Power coefficient in the power function $k_{\mathrm{sr}}\left( \theta\right)$ when decreasing |  |  |
| *θ* | V | Soil water content | m^3^ m^-3^ |  |
| *θ** | P | Optimal soil water content | m^3^ m^-3^ |  |
| *θ*_res_, *θ*_sat_ | C | Residual and saturated soil water content, respectively | 0.098 m^3^ m^-3^,  0.918 m^3^ m^-3^ | Päivänen (1973) |
| *ι* | P | Initial slope of irradiance response curve | m^3^ mol^-1^ |  |
| *λ* | P | Marginal carbon gain per water cost | mol CO_2_ mol^-1^ H_2_O |  |
| *ξ*_m_ | Pe | Multiplier coefficient in the power function $k_{\mathrm{sr}}\left( \theta\right)$ when increasing | mol m^-2^ s^-1^ Pa^-1^ |  |
| *ξ*_p_ | Pe | Power coefficient in the power function $k_{\mathrm{sr}}\left( \theta\right)$ when increasing |  |  |
| *ρ* | C | (All-sided) Leaf-sapwood areas ratio | 2500 m^2^ leaf m^-2^ sapwood | Whitehead (1978); Flower-Ellis and Olsson (1993). |
| *τ* | C | Time constant in the photosynthetic acclimation of foliage to temperature | 12 d | Mäkelä et al (2004) |
| Ψ_s_, Ψ_sat_ | V | Soil water potential and its saturated value, respectively | Pa |  |
| $\mathcal{N}$ |  | Probability density function of the normal (Gaussian) distribution |  |  |
| **W_1_**, **W_2_** |  | Tree groups (i.e. sets of *i*) due to forms of heteroscedasticity in the data model |  |  |

† Type: C, constant throughout the study; P, parameter not directly estimated in the study (but as functions or components of others); Pe, parameter directly estimated in the process or data model; S, subscript; V, variable without direct observations; Vo, variable directly observed; (blank), not applicable.

**References of Supplementary Information**

Flower-Ellis JGK, Olsson L (1993) Estimation of volume total and projected area of Scots pine needles from their regression on length. Stud For Suec 190:1-19.

Hölttä T, Lintunen A, Chan T, Mäkelä A, Nikinmaa E (2017) A steady-state stomatal model of balanced leaf gas exchange hydraulics and maximal source-sink flux. Tree Physiol 37(7):851-868.

Martínez‐Vilalta J, Cochard H, Mencuccini M, Sterck F, Herrero A, Korhonen JFJ, Llorens P, Nikinmaa E, Nolè A, Poyatos R, Ripullone F, Sass-Klaassen U, Zweifel R (2009) Hydraulic adjustment of Scots pine across Europe. New Phytol 184(2):353-364.

Mäkelä A, Hari P, Berninger F, Hänninen H, Nikinmaa E (2004) Acclimation of photosynthetic capacity in Scots pine to the annual cycle of temperature. Tree Physiol 24(4):369-376.

Nikinmaa E, Hölttä T, Hari P, Kolari P, Mäkelä A, Sevanto S, Vesala T (2013) Assimilate transport in phloem sets conditions for leaf gas exchange. Plant Cell Environ 36(3):655-669.

Päivänen J (1973) Hydraulic conductivity and water retention in peat soils. Suomen Metsätieteellinen Seura, Helsinki, Finland.

Whitehead D (1978) The estimation of foliage area from sapwood basal area in Scots pine. Forestry 51(2):137-149.
